# Guardian Presence in Research Conducted on Children With Blunt Abdominal Trauma in the Emergency Department

**DOI:** 10.1111/acem.70308

**Published:** 2026-05-06

**Authors:** Irma T. Ugalde, Mohamed Badawy, Kevan A. McCarten‐Gibbs, Kenneth Yen, Paul Ishimine, Nisa S. Atigapramoj, Pradip P. Chaudhari, Daniel J. Tancredi, Nathan Kuppermann, James F. Holmes

**Affiliations:** ^1^ Department of Pediatrics University of Chicago Pritzker School of Medicine Chicago Illinois USA; ^2^ Department of Pediatrics UT Southwestern Dallas Texas USA; ^3^ Department of Emergency Medicine UCSF Benioff Children's Hospital Oakland California USA; ^4^ Departments of Emergency Medicine and Pediatrics University of California, San Diego School of Medicine La Jolla California USA; ^5^ Division of Emergency and Transport Medicine, Keck School of Medicine of the University of Southern California Los Angeles California USA; ^6^ Department of Pediatrics UC Davis School of Medicine Sacramento California USA; ^7^ Departments of Pediatrics and Emergency Medicine George Washington University School of Medicine and Health Sciences, Children's National Hospital Washington DC USA; ^8^ Department of Emergency Medicine UC Davis School of Medicine Sacramento California USA

Trauma is the leading cause of death and life years lost in children [1] making research to determine best practices critical. The National Institutes of Health underscores this by calling for children to be included in all human subjects' research unless compelling or ethical reasons not to include them exist. As their participation is contingent on the moral and legal obligations to protect children who are unable to provide informed, reasoned, and voluntary consent to their participation, most research in children requires parental permission. Parental presence may not always be possible in emergency situations which can limit timely research. As a pathway for emergency clinical research, the Food and Drug Administration (FDA) created the exception from informed consent (EFIC) pathway. This pathway has strict criteria, requires extensive community consultation and disclosure, and includes stipulations to seek informed consent as soon as possible [2]. It is important to understand how these competing forces potentially impact pediatric research conducted in emergency settings. In this secondary analysis of a large prospective multicenter study, we set out to examine the frequency of guardian presence at the initial emergency department (ED) evaluation of children with blunt abdominal trauma and explored the potential impact on research of this population if parental permission had been required.

This was a planned secondary analysis of a large, prospective multicenter observational study conducted at six EDs, all serving as Level 1 Pediatric Trauma Centers. The UC Davis institutional review board (IRB) approved the study, and all other sites relied on this approval. Children (< 18 years) sustaining blunt abdominal trauma were eligible. Details of the primary study such as inclusion/exclusion criteria are published elsewhere [3, 4]. ED providers completed standardized case report forms on all patients with information pertinent to the primary study objective including mechanism of injury, the presence of the PECARN abdominal injury decision rule variables, and the presence/absence of a guardian along with reasons for absence. We collected patient demographics including race, ethnicity, and the social deprivation index (SDI), a measure of the level of socioeconomic disadvantage calculated from the zip code. Such variables were included to better understand associated characteristics of children at most risk for unanticipated exclusion of research requiring guardian permission. This is pertinent because parents with low socioeconomic status or belonging to a racial or ethnic minority have been associated with lower enrollments in pediatric trials [5]. Patients were excluded for the present study if the providing clinicians failed to document the presence or absence of the guardian at the time of the initial ED evaluation. The questions regarding guardian presence or absence were added to the case report form after the original study had already started. These questions were added to robustly analyze reports of guardian absence. The primary outcome was presence or absence of a guardian at the time of initial ED evaluation. Secondary outcomes included reasons for guardian absence and time of guardian arrival to the ED. Data are described with simple descriptive statistics including 95% exact (Clopper–Pearson) confidence intervals (CI). Patient variables associated with guardian absence in univariable analyses were then entered into a multiple logistic regression analysis using maximum likelihood estimation controlling for site to identify those variables independently associated with guardian absence. These results are reported as adjusted odds ratios (aOR) with 95% CIs. Data analysis was performed with Stata version 19.0 (StataCorp 2025, College Station, Texas 77845, USA).

A total of 2406 eligible patients presented between March 2020 and August 2021. Of these, 2387 (99.2%, 95% CI 98.8%, 99.5%) had guardian presence documented. The mean patient age was 9.4 ± 5.0 years and 1356 (57%) were male. Patient characteristics are presented in Table 1, stratified by guardian presence or absence. 769 (32%) of the total sampled had guardian absence. Multivariable analysis identified several variables independently associated with guardian absence including increasing patient age (aOR 1.09, 95% CI 1.11, 1.7), motor vehicle collision (aOR 1.96, 95% CI 1.49, 2.59), Glasgow Coma Scale GCS score < 14 (aOR 4.87, 95% CI 3.46, 6.86), thoracic wall trauma (aOR 1.47, 95% CI 1.13, 1.92), and absent/decreased breath sounds (aOR 4.12, 95% CI 1.72, 9.87) whereas falls were associated with guardian presence (aOR 0.65, 95% CI 0.44, 0.96). Of the 769 patients with guardian absence, 606 (79%) had reasons documented. Absent caregivers were frequently involved in the same trauma and were being cared for at the same (17.3%) or different ED (13.9%) or died in the trauma (0.4%). The frequency of guardian absence varied across study sites (15.1%, 16.7%, 19.9%, 26.7%, 40.6% and 52%). Time of guardian arrival was collected in 281 (36.5%, 95% CI 33.1, 40.1%) patients who did not have a guardian initially present, and the median time for the guardian to arrive was 66 (IQR 31, 119) minutes.

**TABLE 1:** Patient characteristics stratified by guardian presence and guardian absence.

	Guardian present (*n* = 1618)	Guardian absent (*n* = 769)	Difference in means or rates (95% CIs)
Age (mean ± SD) years	8.8 ± 4.9	10.6 ± 5.0	1.8 (1.4, 2.2)
Sex (male)	910 (56%)	446 (58%)	−2% (−6%, 2%)
Race/Ethnicity			
American Indian or Alaska Native	7 (0.4%)	3 (0.4%)	0 (−0.5%, 0.6%)
Asian	33 (2.0%)	13 (1.7%)	0.3% (−0.8%, 1.5%)
Black	399 (24.7%)	198 (25.7%)	−1.1% (−4.8%, 2.6%)
Native Hawaiian or Pacific Islander	2 (0.1%)	1 (0.1%)	0.0 (−0.3%, 0.3%)
White	891 (55.1%)	374 (48.6%)	6.4% (2.1%, 10.7%)
Unknown	122 (7.5%)	90 (11.7%)	−4.1% (−6.8%, −1.6%)
Other/multiple	164 (10.1%)	95 (12.4%)	−2.2% (−5.0%, 0.5%)
Ethnicity (Hispanic)	704 (44.0%)	290 (38.4%)	5.7% (1.4%, 9.9%)
Social Deprivation Index score			
1–25	200 (12.4%)	107 (14.0%)	−1.6 (−4.5%, 1.4%)
26–50	234 (14.5%)	126 (16.4%)	−1.9 (−5.1%, 1.2%)
51–75	292 (18.1%)	145 (18.9%)	−0.1 (−4.2%, 2.5%)
76–100	890 (55.1%)	389 (50.7%)	4.4% (0.1%, 8.6%)
Mechanism of injury			
Motor vehicle collision	733 (45%)	496 (64%)	−19% (−23%, −15%)
Auto versus pedestrian	135 (8.3%)	60 (7.8%)	0.5% (−1.8%, 2.9%)
Motorized vehicle	236 (14.6%)	95 (10.9%)	3.7% (0.1%, 6.3%)
Fall (elevation)	241 (14.9%)	42 (5.5%)	9.4% (7.1%, 11.8%)
Fall (ground)/run into object	31 (1.9%)	16 (2.1%)	−0.2% (−1.4%, 1.0%)
Fall (stairs)	25 (1.5%)	1 (0.1%)	1.4% (0.8%, 2.1%)
Bicyclist	92 (5.7%)	27 (3.5%)	2.2% (0.4%, 3.9%)
Assault	17 (1.1%)	7 (0.9%)	0.1% (−0.7%, 1.0%)
Object struck abdomen	35 (2.2%)	5 (0.7%)	1.5% (0.6%, 2.4%)
Fall out vehicle	11 (0.7%)	3 (0.4%)	0.3% (−0.3%, 0.9%)
Crush	11 (0.7%)	2 (0.3%)	0.4% (−0.1%, 1.0%)
Sports	8 (0.5%)	2 (0.3%)	0.2% (−0.3%, 0.7%)
Other/unknown	45 (2.8%)	13 (1.7%)	1.1% (−0.1%, 2.3%)
Physical examination findings			
Abdominal pain	322 (20.2%)	148 (19.4%)	0.8% (−2.6%, 4.2%)
Vomiting	123 (7.7%)	63 (8.3%)	−0.6% (−2.9%, 1.8%)
Absent/decreased breath sounds	8 (0.5%)	29 (3.8%)	−3.3% (−4.7%, −1.9%)
Evidence of thoracic wall trauma	218 (13.7%)	169 (22.1%)	−8.5% (−11.9%, −5.1%)
Evidence of abdominal wall trauma	285 (17.9%)	165 (21.6%)	−3.7% (−7.1%, −0.2%)
Abdominal tenderness	324 (20.3%)	169 (22.1%)	−1.8% (−5.4%, 1.7%)
Glasgow Coma Scale score < 14	74 (4.6%)	132 (17.3%)	−12.7% (−15.5%, −9.8%)
ED abdominal CT	362 (22.4%)	391 (49.2%)	−26.8% (−30.9%, −22.7%)
Hospital admission	545 (33.7%)	412 (53.6%)	−19.9% (−24.1%, −15.7%)
Intra‐abdominal injury	69 (4.3%)	95 (12.4%)	−8.1% (−10.6%, −5.5%)
Intra‐abdominal injury undergoing acute intervention	14 (0.9%)	21 (2.7%)	−1.9% (−3.1, −0.6)
Blood transfusion for abdominal bleeding	4 (0.2%)	11 (1.4%)	−1.2 (−2.1%, 0.3%)
NPO for ≥ 2 nights (Pancreas/GI injury)	10 (0.6%)	11 (1.4%)	−0.8% (−1.7, 0.1)
Laparotomy	7 (0.4%)	14 (1.8%)	−1.4 (−2.4%, −0.4%)

*Note:* Ethnicity was missing in 32 cases. Social Deprivation Index is a composite measure of seven demographic characteristics based on the patient's zip code that describes patient hardship, with higher scores equating to more deprivation. It was missing in four cases. Motorized vehicle includes motorcycle, all‐terrain vehicles, and other motorized scooters.

Abbreviations: CI, confidence interval; ED, emergency department; GI, gastrointestinal; IAI, Intra‐abdominal injury; NPO, nothing by mouth; SD, standard deviation.

In summary, one third of children with blunt abdominal trauma did not have a guardian present during their initial ED evaluations. Patients whose guardians were absent were older, more severely injured, and more likely to have intra‐abdominal injuries. While they were more likely to be injured in motor vehicle collisions and have GCS scores < 14, they were less likely to have been injured in falls.

The higher association of older children with guardian absence at the time of ED presentation is understandable as older children are more independent and may be farther away from guardians when they are injured. For example, they may be involved in sports, school activities, and play away from home with less parental supervision. An expected time lapse is reasonable until guardians are notified and can meet their children in the ED. Patients without guardians present were more severely injured with higher odds of having GCS scores < 14 and higher percentages with abdominal injuries undergoing acute interventions than those whose guardians were present. Severely injured children are emergently transported to EDs frequently without their guardians [6].

Our findings have potential implications for research involving acutely injured children. The primary study was observational and did not require guardian permission for patient enrollment. If a similar patient cohort enrolled in research required guardian permission, it is likely that the guardian absence could bias the findings by excluding older and more severely injured children. This selection bias could prohibit enrolling a representative study population and producing generalizable results. In addition, if IRB policy requires the guardian to be physically present to provide permission, such delays may preclude timely enrollment in time‐sensitive research, given that more than half of guardians not present initially arrived more than 1 h after the child's ED presentation. A previous study investigating the use of TXA in severely injured children required parental permission for enrollment; however, the study investigators were unable to enroll sufficient participants because parents were frequently absent during the initial evaluation [7]. Subsequently, when EFIC was granted and instituted, patient enrollment was successful [7]. These practical consequences must be considered when determining regulations for pediatric trauma research conducted in the ED, recognizing not all important research conducted in the ED will meet EFIC requirements.

Similar to our findings, a prior single‐center observational study on children with nontrivial head trauma conducted by our group demonstrated that nearly half (45%) of children did not have guardians available during the initial ED evaluation [6]. Interestingly, that study demonstrated similar independent associations between children whose guardians were not present and certain factors identified in the current study (higher likelihood of motor vehicle collisions, GCS scores < 14, and older children), likely for the same reasons as outlined above. In another multicenter study involving pediatric intensive care units, 35% of patients eligible for research did not have legal guardians at the bedside on the first attempted encounter for permission [8]. One quarter of approached patients were ultimately not enrolled due to the inability to contact the guardian during the hospital stay or failure of a permission decision by the child's legal guardian before discharge. This demonstrates the degree of potential loss of enrollments in studies of acutely ill and injured children and the possibility to introduce bias into these studies.

The current study had limitations. It was conducted at academic, referral, children's hospitals serving as specialized trauma centers in metropolitan regions across the United States although similar patient groups will present at many community and rural hospitals. While we investigated the presence of guardians at the bedside, it is unknown if it was possible to connect with guardians before their arrival as this information was not specifically sought. Furthermore, the time of guardian arrival was documented for only 36.5% of children without guardians present at initial evaluation, likely reflecting challenges that clinicians faced in determining when guardians arrived.

One third of children with blunt abdominal trauma did not have guardians available during their initial ED evaluations. This absence may prolong time to enrollment, impact sample size attainment, and contribute to enrollment bias in pediatric trauma research. These factors can significantly contribute to the success of a study and its ability to answer the central question posed.

## Author Contributions

Dr. Nathan Kupperman conceived the study, designed the trial, obtained research funding, recruited participating centers, managed the data and quality control, and critically reviewed and revised the manuscript for important intellectual content. Dr. James F. Holmes conceived the study, designed the trial, obtained research funding, recruited participating centers, managed the data and quality control, provided statistical analysis, and critically reviewed and revised the manuscript for important intellectual content. Drs. Ken Yen, Mohamed Badawy, Kevan McCarten‐Gibbs, Paul Ishimine, Nisa S. Atigapramoj, and Pradip P. Chaudhari supervised the conduct of the trial and data collection and edited the manuscript. Daniel Tancredi provided statistical advice on study design and analyzed the data. Dr. Irma Ugalde supervised the conduct of the trial and data collection, analyzed the data, and drafted the initial manuscript. *All authors approved the final manuscript as submitted and agree to be accountable for all aspects of the work*.

## Funding

This work was supported by Eunice Kennedy Shriver National Institute of Child Health and Human Development (Grant R01HD084674).

## Conflicts of Interest

The authors declare no conflicts of interest.

## Data Availability

The data for this study will be made available for qualified researchers with the execution of a Data Sharing Agreement.
